# Effects of cycloheximide on the interpretation of ribosome profiling experiments in *Schizosaccharomyces pombe*

**DOI:** 10.1038/s41598-017-10650-1

**Published:** 2017-09-04

**Authors:** Caia D. S. Duncan, Juan Mata

**Affiliations:** Department of Biochemistry, University of Cambridge, Cambridge, CB2 1QW United Kingdom

## Abstract

Stress conditions lead to global and gene-specific changes in RNA translation. Ribosome profiling experiments have identified genome-wide alterations in the distribution of ribosomes along mRNAs. However, it is contentious whether these changes reflect real responses, or whether they are artefacts caused by the use of inhibitors of translation (notably cycloheximide). To address this issue we performed ribosome profiling with the fission yeast *Schizosaccharomyces pombe* under conditions of exponential growth (unstressed) and nitrogen starvation (nutritional stress), and both in the presence and absence of cycloheximide. We examined several aspects of the translational response, including density of ribosomal footprints on coding sequences, 5′ leader ribosomal densities, distribution of ribosomes along coding sequences, and ribosome codon occupancies. Cycloheximide had minor effects on overall ribosome density, which affected mostly mRNAs encoding ribosomal proteins. Nitrogen starvation caused an accumulation of ribosomes on 5′ leaders in both cycloheximide-treated and untreated cells. By contrast, stress-induced ribosome accumulation on the 5′ side of coding sequences was cycloheximide-dependent. Finally, codon occupancy showed strong positive correlations in cycloheximide-treated and untreated cells. Our results demonstrate that cycloheximide does influence some of the results of ribosome profiling experiments, although it is not clear if this effect is always artefactual.

## Introduction

Ribosome profiling has revolutionized the study of translation by providing a genome-wide, single-nucleotide resolution view of this process^1^. The approach is based on the property that mRNA-bound ribosomes protect a small mRNA fragment from degradation by nucleases^2, 3^. Treatment of cell extracts with a ribonuclease (typically RNase I) leads to the degradation of unprotected mRNA. Ribosome-protected fragments (RPFs or ribosome footprints) are then isolated and processed for analysis by high-throughput sequencing^1^. Ribosome profiling can be used to estimate overall translation rates as well as asymmetries in the distribution of ribosomes on mRNAs^4, 5^.

The key assumption of ribosome profiling is that the distribution of ribosomes on mRNAs at the time of RNase digestion faithfully reproduces their location *in vivo*. To ensure that this is the case, numerous studies have used inhibitors of translation elongation, typically cycloheximide (CHX), to ‘freeze’ ribosomes in their *in vivo* distribution^6, 7^. However, a number of recent studies, mostly using the budding yeast *Saccharomyces cerevisiae*, have brought into question the use of CHX in ribosome profiling experiments^8–10^. For example, Lareau *et al*. reported that ribosomes protect two populations of mRNA fragments of different sizes, each of which is stabilised by a different translation inhibitor. Importantly, the distribution of these reads on individual codons was uncorrelated between both subpopulations^10^.

Several papers using *S. cerevisiae* reported a rise of ribosome footprints in 5′ leader sequences upon stress conditions, suggesting higher use of translated upstream Open Reading Frames (uORFs) in response to stress^1, 11, 12^. However, a comprehensive investigation that examined the effect of several concentrations of CHX on ribosome distribution upon stress concluded that these observations were due to artefactual, CHX-induced rearrangements^8^.

Other studies that used CHX reported a broad accumulation of ribosomal footprints on the 5′ side of coding sequences in *S. cerevisiae* (in the ~300 initial nucleotides), which was strongly increased by amino acid starvation and oxidative stress^1, 12^ and that was not observed in mammalian cells^13^. A detailed examination of this phenomenon in *S. cerevisiae* showed that this peak was smaller both in cells untreated with CHX and in those preincubated with very high concentrations of the drug^8^. Moreover, the increases caused by oxidative stress, heat shock and amino acid starvation were found to be CHX-dependent^8^. This suggested a model in which intermediate concentrations of the drug are slow to act (possibly due to limiting diffusion); as translation initiation is not inhibited, newly-initiating ribosomes would continue translating until they encounter the drug, thus artefactually increasing ribosome density in the first few hundred nucleotides of the coding sequence^8^. However, a broad peak of ribosome density at the 5′ of coding sequences is clearly present in unstressed cells that have not been treated with CHX^1, 14^, although it is only apparent when cells are flash-frozen^1^.

In mammalian tissue culture cells incubated with CHX, ribosome profiling has revealed that heat shock and proteotoxic stress cause arrest/pausing of translational elongation at around codon 65^15, 16^. It was also found that both treatments lead to increase ribosome density in 5′ leaders. The dependence on CHX of these two phenotypes has not been examined.

Finally, ribosome profiling has been used to investigate the relative distribution of ribosomes on individual codons, which is predicted to reflect codon-specific translation speeds^17–20^. A recent study re-examined multiple ribosome profiling experiments (performed in *S. cerevisiae* in the absence or presence of CHX) to investigate codon-specific ribosome occupancies^9^. The authors concluded that translation elongation can continue for a few codons in the presence of CHX, although with codon-specific unphysiological rates, leading to artefactual distributions of ribosomes^9^.

The majority of the above studies were performed in *S. cerevisiae*, and thus it is unclear to what extent their conclusions are applicable to other species. However, this is an essential question for every organism used for ribosome profiling, both for the design of future experiments and for the interpretation of those already completed in the presence of CHX.

We have examined the effect of CHX on ribosome profiling experiments performed with the fission yeast *Schizosaccharomyces pombe*, a commonly used unicellular model organism^21^. We compared cells growing vegetatively in the presence of a nitrogen source with cells starved for nitrogen (nutritional stress). Nitrogen starvation in *S. pombe* activates a rapid and complex transcriptional programme^22, 23^, but the translational response that accompanies this process has not been examined. We carried out ribosome profiling experiments (together with standard RNA-seq) in the presence or absence of CHX, and focused on four aspects of translation: 1] Total ribosome density for coding sequences of individual genes (which is a proxy for how much they are translated), 2] occupancy of 5′ leader sequences, 3] biases in ribosome distribution across coding sequences and 4] distribution of ribosomes on individual codons (expected to represent codon-specific translation rates). Our results reveal that CHX affects some, but not all, of these parameters. However, it is still unclear if all of these effects are artefactual, or if in some cases cycloheximide can help preserve the physiological distribution of ribosomes on some mRNAs.

## Results and Discussion

### Experimental design and reproducibility

To examine the effects of CHX on ribosome profiling experiments we applied this technique to *S. pombe* cells growing exponentially (unstressed) and after 1 hour of nitrogen starvation (nutritional stress). Each culture was split into two, and one of them was incubated with CHX at a concentration of 100 µg/ml for 5 minutes before collection (this is the ‘standard′ concentration used in the majority of published experiments). Cells were collected by filtration and immediately flash-frozen in liquid nitrogen to prevent further translation. Note that CHX was present in lysis buffers for all samples. Thus, all references to CHX treatment below apply only to its addition to the culture medium. We performed two independent biological replicates for each of the four experiments (plus/minus nitrogen, plus/minus CHX). For each sample, we prepared and isolated ribosome-protected fragments (RPFs or ribosome footprints) as described in Methods, and analysed them using high throughput Illumina sequencing. We also sequenced rRNA-depleted RNA from each of the eight samples (RNA-seq). To evaluate the reproducibility of the technique we quantified for each experiment the number of RPF and RNA-seq reads that mapped to each annotated coding sequence of the *S. pombe* genome. The data were highly reproducible, with average correlations between independent biological replicates of 0.97 (Table 1). We focus below on how CHX affects ribosome profiling experiments. A more complete analysis of the biology of the response of *S. pombe* cells to nitrogen starvation will be published elsewhere.

**Table 1:** Correlation between independent replicates.

Experiment	Correlation
+nitrogen/+CHX RPFs	0.989
+nitrogen/−CHX RPFs	0.990
−nitrogen/−CHX RPFs	0.969
−nitrogen/+CHX RPFs	0.972
+nitrogen/+CHX mRNA	0.958
+nitrogen/−CHX mRNA	0.934
−nitrogen/−CHX mRNA	0.944
−nitrogen/+CHX mRNA	0.931
+nitrogen/+CHX TE	0.956
+nitrogen/−CHX TE	0.916
−nitrogen/−CHX TE	0.923
−nitrogen/+CHX TE	0.928

To investigate the effects of CHX we examined four aspects of translation: 1] Total ribosomal density for coding sequences of individual genes, 2] presence of ribosomes on 5′ leader sequences, 3] biases in ribosome location across coding sequences and 4] distribution of ribosomes along individual codons.

### Ribosome density

**Figure 1:**
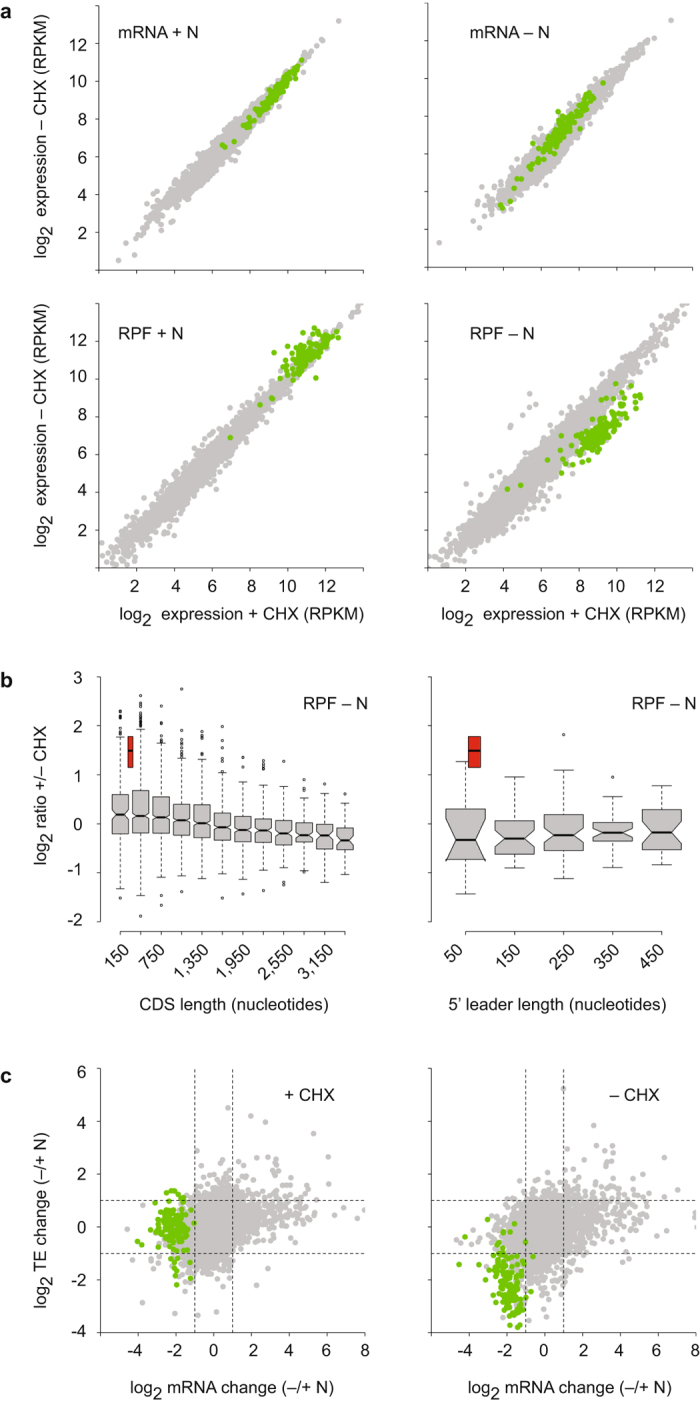
Effects of CHX on overall ribosome densities on coding sequences. (**a**) Scatter plots comparing mRNA levels (top) and ribosome densities (bottom) between untreated and CHX-treated cells. Data are presented for cells growing in the presence of a nitrogen source (+N) or starved for nitrogen (−N). Genes encoding ribosomal proteins are shown in green. All data have been normalized to RPKMs (Reads Per Kilobase per Million mapped reads). (**b**) Left: Boxplots comparing ribosomal densities between untreated and CHX-treated cells for groups of genes of the indicated average lengths of coding sequences. Right: similar data for indicated lengths of 5′ leader sequences. The red boxes display the behaviour of mRNAs encoding ribosomal proteins. (**c**) Comparison of changes in mRNA levels and ribosomal densities between cells starved for nitrogen (−N) and unstressed cells (+N). Data are presented for CHX-treated cells (left) and for untreated cells (left). Genes encoding ribosomal proteins are shown in green.

We quantified the number of RPF reads on coding sequences for each annotated gene in cells treated with CHX or mock-treated. Under nitrogen-starvation conditions the correlation between both treatments was very high (average *R* = 0.96), but ~4.5% of all genes showed consistently higher ribosomal density in the presence of CHX (2-fold or higher in both replicates, Fig. 1a and Supplementary Fig. S1). Similar changes were not observed in mRNA samples (average *R* = 0.98), indicating that this effect was due to alterations in translation and not in the transcriptome (Fig. 1a, Supplementary Fig. S1).

Surprisingly, this group included most genes encoding ribosomal proteins (RPs, Fig. 1a and Supplementary Fig. S1, green dots). To rule out the possibility that CHX causes subtle alterations in mRNA levels, we compared the changes induced by nitrogen starvation in the presence or absence of CHX (Supplementary Fig. S2). The median fold-change in mRNA levels for RP genes was 0.25 in CHX-treated samples and 0.24 in untreated cells, confirming that alterations in ribosomal density of RP genes upon nitrogen starvation are due to changes in translation.

RP genes are generally quite short, with a median length of 447 nucleotides compared to 1,131 for all genes. Therefore, a simple explanation for this enrichment could be that CHX prevents ribosome ‘run-off’ from short genes during cell collection, thus increasing their apparent ribosome density. However, although there was a small trend towards ribosomal density in shorter genes to be higher in the presence of CHX, this was a minor effect and could not account for the behaviour of ribosomal protein genes (Fig. 1b). The mRNAs encoded by these genes also tend to have shorter 5′ leader sequences (a median of 68.5 nucleotides versus 173 for all genes), but there was no overall correlation between 5′ leader lengths and higher ribosome densities in CHX (Fig. 1c). Only 9 nuclear-encoded genes (~0.2%) showed a reduction in ribosome densities in CHX (Fig. 1a and Supplementary Fig. S1), and they lacked any common features.

By contrast, under non-stress conditions, the drug had a very weak effect on ribosomal density (average *R* = 0.98), with less than 1% of genes showing differences in density higher than 2-fold (23 genes higher in CHX and 9 lower, Fig. 1a and Supplementary Fig. S1). Interestingly, the small group of genes that displayed lower densities in cells treated with CHX was also enriched in ribosomal protein genes (12/23 mRNAs).

We conclude that mRNAs encoding RPs are particularly sensitive to the presence of CHX, and that this phenomenon cannot be explained solely by their short 5′ leader and coding sequences. In addition, the effect is only strong under nutritional stress. However, these results do not reveal which of the two samples (CHX-treated or -untreated) reflects better the *in vivo* situation. For example, RP genes are rich in optimal codons, implying that translation elongation occurs at high speed. This property, together with their short length, might make them more sensitive to ribosome run-off during collection. In this case, CHX would stabilise the *in vivo* distribution. Alternatively, it is also possible that CHX has a direct effect on the translation of these mRNAs, leading to unphysiological ribosomal densities.

We then examined whether the changes in ribosome density caused by CHX would affect the interpretation of the translational/transcriptional response to nitrogen starvation. We quantified translational efficiencies (TEs) by normalising RPF counts by mRNA levels, and calculated the log change in TE and transcript levels between cells grown in nitrogen-containing medium and nitrogen-starved cells (Fig. 1c and Supplementary Fig. S1B). In the presence of CHX, nitrogen starvation led to a clear down-regulation in the levels of mRNAs encoding RPs, but did not affect their TE. By contrast, in experiments performed in the absence of CHX these mRNAs appeared to be down-regulated both at the mRNA and the TE levels. Thus, pre-incubation with CHX in the medium can affect the TE of specific groups of genes. The abundance of mRNAs encoding RPs is very tightly co-regulated^22–24^; our results demonstrate that these mRNAs also show coordinated behaviour at the translational efficiency level. The reason for the extreme sensitivity of these mRNAs to CHX remains to be clarified.

For the majority of genes, though, treatment with CHX has no effect on ribosomal density regardless of the growth condition. Similar results have been reported for mammalian cells grown in culture, with CHX having no significant effect on gene-specific ribosome densities. However, this has only been examined in unstressed cells^13^.

### Changes in the use of upstream Open Reading Frames

*S. cerevisiae* cells display an accumulation of ribosome footprints in 5′ leader sequences that is increased under conditions of stress, suggesting higher usage of uORFs^1, 9, 10^. However, these conclusions have been disputed and attributed to the use of CHX in cell culture^8^.

**Figure 2:**
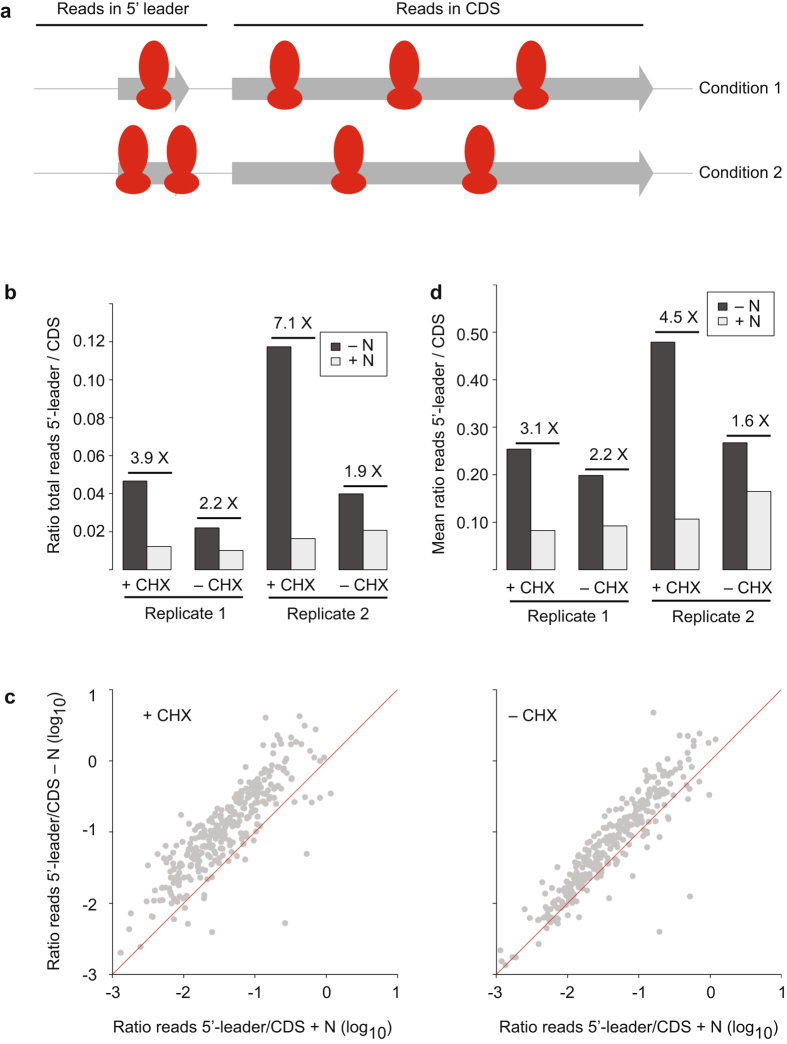
Effects of CHX on ribosome densities on 5′ leader sequences. (**a**) Experimental design: RPFs in coding sequences (CDS) and 5′ leader sequences are quantified in different experimental conditions. (**b**) Ratio of total reads mapping to 5′ leader sequences to total reads mapping to coding sequences (CDS) for unstressed cells (+N) and nitrogen starved cells (−N), and for cells treated or untreated with CHX (±CHX). The numbers indicate the fold-difference between pairs of −N and +N samples. Data are presented for two biological replicates. (**c**) Scatter plot comparing the ratio of reads mapping to 5′ leader sequences to reads mapping to coding sequences (CDS) for individual genes; each plot compares unstressed cells (+N) and nitrogen starved cells (−N). Data are presented for cells treated with CHX (left) or untreated (right). The red lines correspond to a ratio of 1. (**d**) Average values for the ratios presented in (**c**). The numbers indicate the fold-difference between pairs of −N and +N samples. Data are displayed for two biological replicates.

To address this question in *S. pombe* we compared the accumulation of reads in 5′ leader and coding sequences (Fig. 2a) before and after nitrogen starvation. We initially quantified this value by measuring the ratio between the total number of ribosome footprints in 5′ leaders and in coding sequences. Nitrogen starvation in CHX-treated cells caused an average increase of 5.5-fold, while untreated cells had a 2.1-fold average increment (both enrichments were consistent across biological replicates, Fig. 2b). As total ratios could be dominated by changes in a small number of highly abundant genes, we also quantified the ratios between footprints in 5′ leaders and coding sequences for all individual transcripts that passed the expression threshold (see Methods for details, and Fig. 2c and Supplementary Fig. S3 for the results). Consistent with the previous result, there was a clear increase in ribosome footprints in 5′ leader sequences upon nitrogen starvation for the majority of genes (Fig. 2c and Supplementary Fig. S3; note the increase in the second replicate is smaller, but still significant), with mean increase ratios of 3.8 and 1.9 for plus/minus CHX, respectively (Fig. 2d, note the similar behaviour of both replicates). Thus, in contrast to the *S. cerevisiae* results, 5′ leader ribosomal densities were increased by nutritional stress in every experiment, although the effect was substantially higher in CHX-treated cells. A possible caveat is, of course, that the *S. cerevisiae* study used a different type of stress^8^. Nevertheless, given that some accumulation is observed both with and without drug treatment, we can conclude that in *S. pombe* nitrogen starvation leads to higher ribosomal densities on 5′ leader sequences. This increase in density may be due to translation of uORFs, although we cannot rule out that it reflects increased noise in stressed samples. Supplementary Fig. S4 presents two examples of uORFs induced in response to nitrogen starvation. The biological importance and mechanistic basis of this phenomenon (as well as whether it is general to all stress conditions) is still unknown.

### Distribution of ribosomes along coding sequences

*S. cerevisiae* cells display an asymmetric ribosome distribution across coding sequences, with a broad peak of higher ribosome occupancy in the initial ~300–400 nucleotides of the coding sequence^1, 9, 14, 20^ that is strongly enhanced by different stresses^1, 8, 12^.

**Figure 3:**
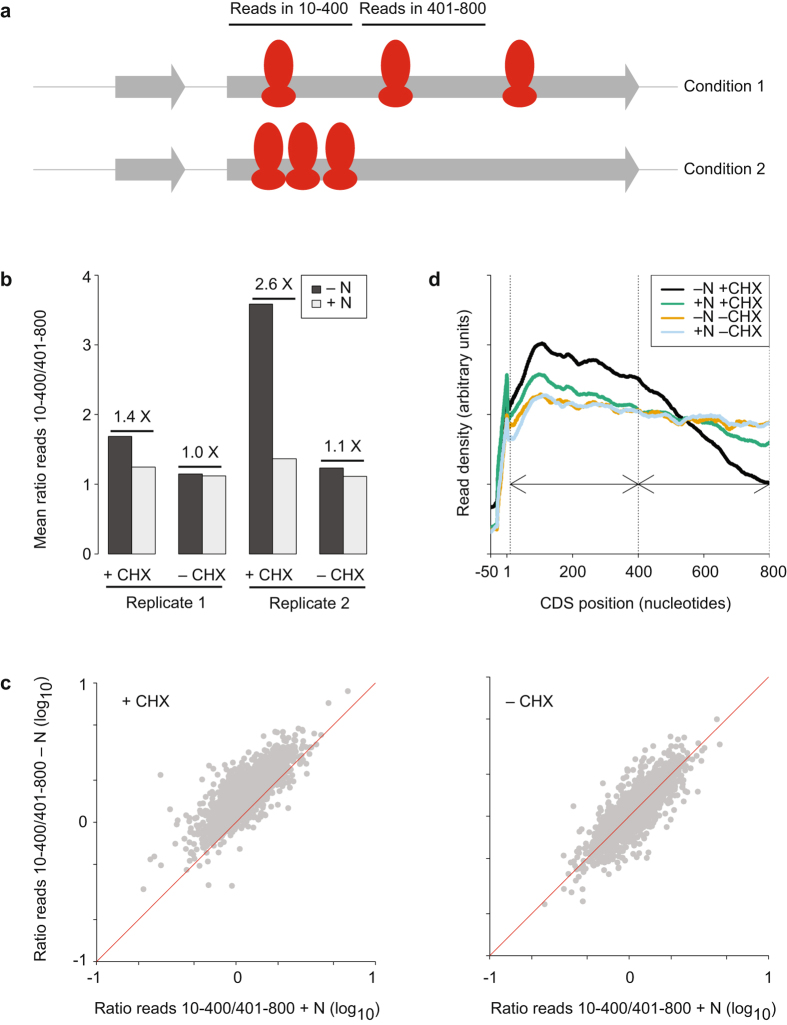
Effects of CHX on ribosome distribution across coding sequences. (**a**) Experimental design: RPFs on nucleotides 10 to 400 and on nucleotides 401–800 are quantified in different experimental conditions, and the ratio between both numbers is calculated. (**b**) Mean ratios calculated as described in A for all coding sequences, in the presence and absence of a nitrogen source (±N) and in the presence and absence of CHX treatment (±CHX). The numbers indicate the fold-difference between paired −N and +N samples. Data are presented for two biological replicates. (**c**) Scatter plot comparing the ratios obtained as defined in A for individual genes; each plot compares unstressed cells (+N) and nitrogen starved cells (−N). Data are presented for cells treated with CHX (left) or untreated (right). The red lines correspond to a ratio of 1. (**d**) Metagene displaying average distributions of RPFs along coding sequences in four experimental conditions. A running window of 60 nucleotides was used to smoothen the plotted lines.

We investigated this phenomenon in *S. pombe* in two ways: first, by calculating the ratio between footprints in nucleotides 10 to 400 and 401 to 800 (Fig. 3a–c, the first 9 nucleotides were not considered to avoid biases created by the accumulation of ribosomes at initiating AUG); second, by examining the behaviour of a metagene representing genome-wide ribosome density along coding sequences (Fig. 3d and Supplementary Fig. S5).

In the absence of stress, *S. pombe* cells showed small shoulders both in the presence and absence of the drug, similar to those reported for *S. cerevisiae* without CHX^1, 14, 20^ (Fig. 3d and Supplementary Fig. S5). Upon nutritional stress, *S. pombe* behaved similarly to *S. cerevisiae* ^12^. In the presence of CHX, there was a clear accumulation of reads in the 5′ part of the coding sequence in the majority of genes (Fig. 3c and Supplementary Fig. S5, left plot), which was also reflected in a ~2.0-fold increase in average density in the first 400 nucleotides of the mRNA (Fig. 3b). By contrast, this increase was negligible in the absence of the drug, both when individual genes were examined (Fig. 3c (right plot) and Supplementary Fig. S5) and when the average ratios were measured (Fig. 3b, note both replicates behaved consistently). Moreover, the CHX-dependence of the accumulation of reads upon nitrogen starvation was confirmed by the metagene data (Fig. 3d and Supplementary Fig. S5). Finally, we established that this effect was specific for RPFs, by plotting a metagene based on mRNA-seq data (Supplementary Fig. S5).

We also considered whether these observations would also apply to smaller genes. With this aim, we generated metagenes for RP genes and for small genes (less than 200 codons) excluding RP genes. In both cases, CHX caused a clear increase on the 5′ side of the coding sequences (especially for RP genes), which was dependent both on nitrogen starvation and CHX treatment (Supplementary Fig. S5).

In addition, there was an accumulation of ribosomes on initiation codons (Supplementary Fig. S6). This feature was already present in untreated cells, although it was elevated with CHX incubation (both in control and nitrogen-starved cells). This enrichment was slightly higher in nitrogen-starved cells, independently of CHX treatment (Supplementary Fig. S6).

Thus, there is a clear accumulation of ribosomes in the initial part of coding sequences, conserved in *S. pombe* and *S. cerevisiae*, and that can be observed both with and without CHX pretreatment. By contrast, the stress-induced enhancement is not observed consistently in the absence of CHX in both yeasts, and thus there is not sufficient evidence to indicate that it occurs *in vivo*. In the future, the use of *in vivo* cross-linking strategies^25^ may help distinguish between these two interpretations.

### Codon occupancy

In principle, the normalised ribosomal occupancy of individual codons is related to the time that the ribosome spends at each codon, and thus can be used to estimate average codon-specific translation rates. However, initial experiments to investigate this phenomenon produced conflicting results^17–20^. An elegant study by Hussmann *et al*., which involved novel experiments, metaanalysis of numerous ribosome profiling experiments from *S. cerevisiae* and mathematical modelling^9^, found that these contradictions could be explained by the effect of CHX on the determination of codon-specific ribosome occupancies. Experiments from different groups performed in the presence of CHX had similar ribosome codon occupancies to one another, as did those carried out without the drug. However, the correlations between CHX- and non-CHX assays were very low. Moreover, experiments with CHX resulted in codon-specific translation rates that showed negative correlations with cognate tRNA abundance, while those that did not include CHX treatment showed the expected positive correlations^9, 20^. Hussmann *et al*. proposed that ribosomes do not stop translation immediately in the presence of CHX in the medium. Instead, translation continues for a few codons with codon-specific translation rates^9^, causing artefactual codon occupancies.

To investigate this phenomenon we analysed codon-specific ribosomal occupancies at A-sites as described in Methods. Briefly, we assigned each read that mapped to a coding sequence to the A-site of a ribosome (that corresponds to nucleotide 16 of a ribosome-protected fragment). We then calculated the normalised occupancy for each codon across the genome (by dividing the frequency with which the ribosome is positioned on each codon by the abundance of the codon on the mRNA). In the absence of biases, this value would be expected to reflect the average time that the ribosome spends at each codon.

**Figure 4:**
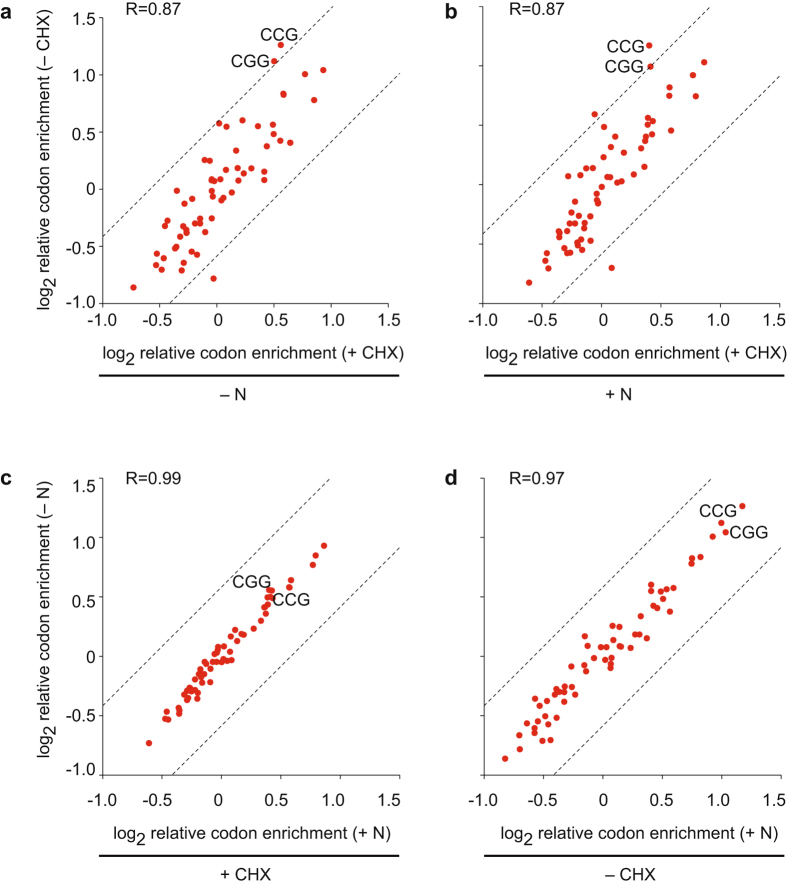
Effects of CHX on relative codon occupancies. Scatter plots displaying relative codon occupancies obtained as described in Methods. Each dot corresponds to a single codon. Termination codons are not displayed. The positions of rare codons CCG and CGG are indicated. The dotted lines correspond to 1.5-fold differences. The Pearson correlations between datasets are indicated. (**a**) Comparison of the effects of CHX treatment in nitrogen-starved cells. (**b**) As in (**a**), for cells grown with a nitrogen source. (**c**) Comparison of the effects of nitrogen starvation in the presence of CHX. (**d**) As in (**c**), in the absence of CHX.

We first compared the effect of CHX on codon-specific ribosomal occupancies (Fig. 4, Supplementary Figs S7 and S8). Surprisingly, the correlation between both experiments was very high, with average values of 0.82 for nitrogen-starvation (Fig. 4a and Supplementary Fig. S7) and 0.86 for unstressed cells (Fig. 4b and Supplementary Fig. S7). In similar comparisons in *S. cerevisiae*, the majority of the correlations were negative^9^. For example, rare codons such as CCG (proline) and CGG (arginine) had amongst the highest occupancies in the absence of CHX, but lost this enrichment in CHX-treated samples^9^. By contrast, in the *S. pombe* dataset both CCG and CGG were enriched regardless of CHX presence (although less strongly in untreated cells, Fig. 4c and Supplementary Fig. S7). Moreover, nutritional stress had very little effect on codon-specific ribosomal occupancies both in the presence (Fig. 4c and Supplementary Fig. S7, average R = 0.96) and in the absence of CHX (Fig. 4d and Supplementary Fig. S7, average R = 0.98).

Finally, we assessed the correlation between tRNA abundance and codon-specific occupancies. This was done by using the tRNA Adaptation Index (tAI), which is a measure of tRNA usage for each codon partly based on tRNA copy number (higher numbers predict more efficient translation)^26^. *S. cerevisiae* experiments performed in the presence of CHX in the culture show negative correlations between the inverse of the tAI (1/tAI) and codon-specific occupancies, predicting that codons with low tAI (and thus low tRNA abundances) would be translated faster. By contrast, experiments with cells not pretreated with CHX showed the expected positive correlation between 1/tAI (although the actual values were very variable among experiments)^9^. In *S. pombe*, we found that in each of the eight ribosome profiling experiments codon-specific occupancies displayed a positive correlation with 1/tAI, with an average of 0.39 (Table 2).

**Table 2:** Correlations between relative codon occupancies and 1/tAI.

	Replicate 1		Replicate 2	
	+N	−N	+N	−N
+CHX	0.35	0.39	0.39	0.28
−CHX	0.40	0.35	0.45	0.49

Our results show that in *S. pombe* CHX has a relatively minor effect on the position of ribosomes on specific codons. This could be explained by *S. pombe* cells being especially sensitive to CHX, so that CHX would block ribosome movements more rapidly and completely than in *S. cerevisiae*. This property would prevent the movement of ribosomes at altered rates (postulated to take place in *S. cerevisiae*), and lead to a situation in which *S. pombe* CHX-treated and untreated cells would have similar ribosome distributions in steady state. However, the fact that rare codons are less enriched in the presence of CHX suggests that CHX should be omitted in experiments aimed at determining codon-specific ribosome distributions.

## Conclusions

We report that in *S. pombe* CHX has only minor effects in overall ribosome density in coding sequences (>95% of genes are unaffected, Fig. 1), similarly to the situation in mammalian cells. This means that CHX could be used to block translation in situations in which flash-freezing is not possible, as long as the experiment is used only for the determination of gene-specific translation rates (albeit with the caveats discussed above for mRNAs encoding RPs). The reason for the high sensitivity of RP mRNAs to CHX, and whether this reflects the endogenous ribosome distribution, remains to be determined. By contrast, care must be taken when the data are used to draw conclusions on the distribution of ribosomes on mRNAs, where we show that the results obtained using treated and untreated cells differ.

Our results suggest that CHX has different effects in *S. pombe* and *S. cerevisiae*. In *S. pombe* cells, but not in *S. cerevisiae*, the stress-induced accumulation of ribosomes in 5′ leader sequences does not require CHX, although it is enhanced by the drug. By contrast, the increased density of ribosomes in the 5′ side of coding sequences is completely dependent of CHX in both organisms. The most striking difference is in the effect of CHX on codon-specific densities: whereas *S. cerevisiae* CHX-treated and untreated cells show negative correlations, drug treatment in *S. pombe* has much smaller effects regardless of nutritional status. However, it is essential to consider that multiple experimental parameters (such as the composition of the lysis buffer, RNase treatment, and library preparation) can influence the results of ribosome profiling experiments^27^. Thus, the results of cross-species comparisons should be interpreted with care.

## Methods

### Strains, growth conditions and experimental design

*S. pombe* cells were grown in Edinburgh Minimal Medium 2 (EMM2) containing 93.4 mM NH_4_Cl at 32 °C. Nitrogen starvation was induced by washing the cells three times in EMM2 without NH_4_Cl (EMM2-N), followed by incubation in EMM2-N for 60 minutes. When indicated, CHX was added to the medium at a concentration of 100 µg/ml, and cells were incubated for 5 minutes. Cells were collected by filtration and immediately frozen in liquid nitrogen (flash-frozen)^28^. All cells were prototrophic with h- mating type. All experiments were performed in duplicate (independent biological replicates performed on different days, with libraries prepared separately).

### Library preparation and sequencing

For RPF analyses, preparation of cell extracts, RNase treatment, separation of samples by centrifugation through sucrose gradients, and isolation of protected RNA fragments were performed as previously described^21^. Gel purified RNA fragments were treated with 10 units of T4 PNK (Thermo Fisher) in a low pH buffer (700 mM Tris pH 7, 50 mM DTT, 100 mM MgCl_2_) for 30 min at 37 °C. ATP and buffer A (Thermo Fisher) were then added for an additional 30 min incubation. RNA fragments were column-purified (PureLink RNA micro-columns, Life Technologies). 100 ng were used as input for the NEXTflex Small RNA Sequencing Kit v2 (Bioo Scientific), and libraries were generated following the manufacturer’s protocol. For mRNA analyses, total RNA was isolated as previously described^21^. rRNA was then removed from total RNA using Ribo-Zero Gold rRNA Removal Kit Yeast (Illumina), with 4 µg as input. Finally, 30 ng of rRNA-depleted RNA was used as starting material for the NEXTflex Rapid Directional qRNA-Seq Kit (Bioo Scientific).

### Data pre-processing and mapping

The structure of the RPF reads is the following: RRRR(NNNN…NNNN)RRRR-adaptor-, where R represents random nucleotides, N corresponds to the sequence of the RNA protected fragment, and the adaptor sequence is TGGAATTCTCGGGTGCCAAGG. Random nucleotides serve as unique molecular identifiers (UMIs)^21^ that allow the removal of PCR duplicates and the generation of a non-redundant dataset. To prepare reads for mapping, adaptor sequences were first removed from the 3′ end of the read. Duplicate reads were then discarded, followed by removal of UMIs. The structure of RNA-seq reads is the following: RRRRRRRRR(NNNN….NNNN), where R(9) corresponds to a UMI and N to the sequence of fragmented RNA molecules. After discarding duplicates reads the RRRRRRRRR sequence was removed from the reads, and reads were reverse complemented before mapping.

Mapping was performed using TopHat 2 version 2.1.1 and Bowtie2 version 2.2.8^29^. For ribosome profiling data, processed reads were first mapped to the *S. pombe* rDNA genome using the following parameters:–read-mismatches 2–no-coverage-search–min-intron-length 29–max-intron-length 819 -z 0 -g 1. Unmapped reads were then aligned to the full *S. pombe* genome with the same settings and with a gff3 file (Schizosaccharomyces_pombe_ASM294v2.28.gff3, downloaded from Ensembl) as a source of information on junctions. For RNA-seq data, processed reads were directly aligned to the *S. pombe* genome using the parameters detailed above.

### Data quantification and statistical analyses

Data quantification (number of reads per coding sequence and 5′ leader sequences and codon occupancies) was carried out using in-house Perl scripts. Only genes with at least ten counts in every sample (8 RPF and 8 mRNA) were used (86% of all annotated coding sequences). All statistical analyses were performed using R^30, 31^. All correlations are Pearson except for the comparisons of ribosomal occupancies to tAI, where Spearman rank correlations were used. All scatter plots shown in the main text correspond to biological replicate number 1. Plots for replicate 2 are presented as supplementary data.

Only genes with at least 10 read counts in every sample (a total of 8 RPF experiments) in both the 5′ leader and coding sequences were used for analysis that involved leader sequences (471 genes, Fig. 2 and Supplementary Fig. S3). The analysis was also performed for the 32 genes that were not detected (less than 10 read counts) in unstressed cells and present in nitrogen-starved samples (data not shown). The results were similar to those in Fig. 2b, although the increase was only seen upon CHX incubation. Note, however, that the low expression levels in these samples makes the calculations of ratios somewhat unreliable.

For the analysis of the distribution of RPFs along coding sequences (Figs 3b,c and Supplementary Fig. S6) only genes with coding sequences longer than 800 nucleotides were used. RPF numbers were quantified for nucleotides 10–400 and 401 to 800. As there is often an enrichment of RPFs in the start codon and nearby nucleotides that may bias the data, nucleotides 1–9 were not used for this comparison. Only genes with at least 24 reads in nucleotides 10–400 (in every experiment) were used. A total of 1,842 genes fulfilled these criteria.

For codon usage analyses RPF reads were aligned to nucleotide 16 (that corresponds to position 1 of the A site). Codons 1–90 were not considered. For each coding sequence the following steps were performed: 1] determination of the fraction of RPFs that occupy each codon (RPFs in a given codon divided by total RPFs); 2] quantification of the relative abundance of each codon on the coding sequence (number of time each codon is present divided by total codon number); 3] define the normalized codon occupancy by dividing parameter 1 by parameter 2. The average codon enrichments were then calculated with data from all coding sequences.

### Data availability

All raw data files have been deposited in ArrayExpress^32^ [http://www.ebi.ac.uk/arrayexpress/] with accession number accession E-MTAB-5794.

## Electronic supplementary material

Supplementary Information
