# Supplementary material for: Effects of cycloheximide on the interpretation of ribosome profiling experiments in Schizosaccharomyces pombe
Source: Sci Rep. 2017 Sep 4;7:10331. doi: 10.1038/s41598-017-10650-1 (PMC5583251; doi:10.1038/s41598-017-10650-1)
Supplement: Supplementary file 1 — Supplementary Information [file 41598_2017_10650_MOESM1_ESM.pdf]

Effects of cycloheximide on the interpretation of ribosome profiling experiments in *Schizosaccharomyces pombe*

Caia D. S. Duncan and Juan Mata

Supplementary information: Figures S1 to S8

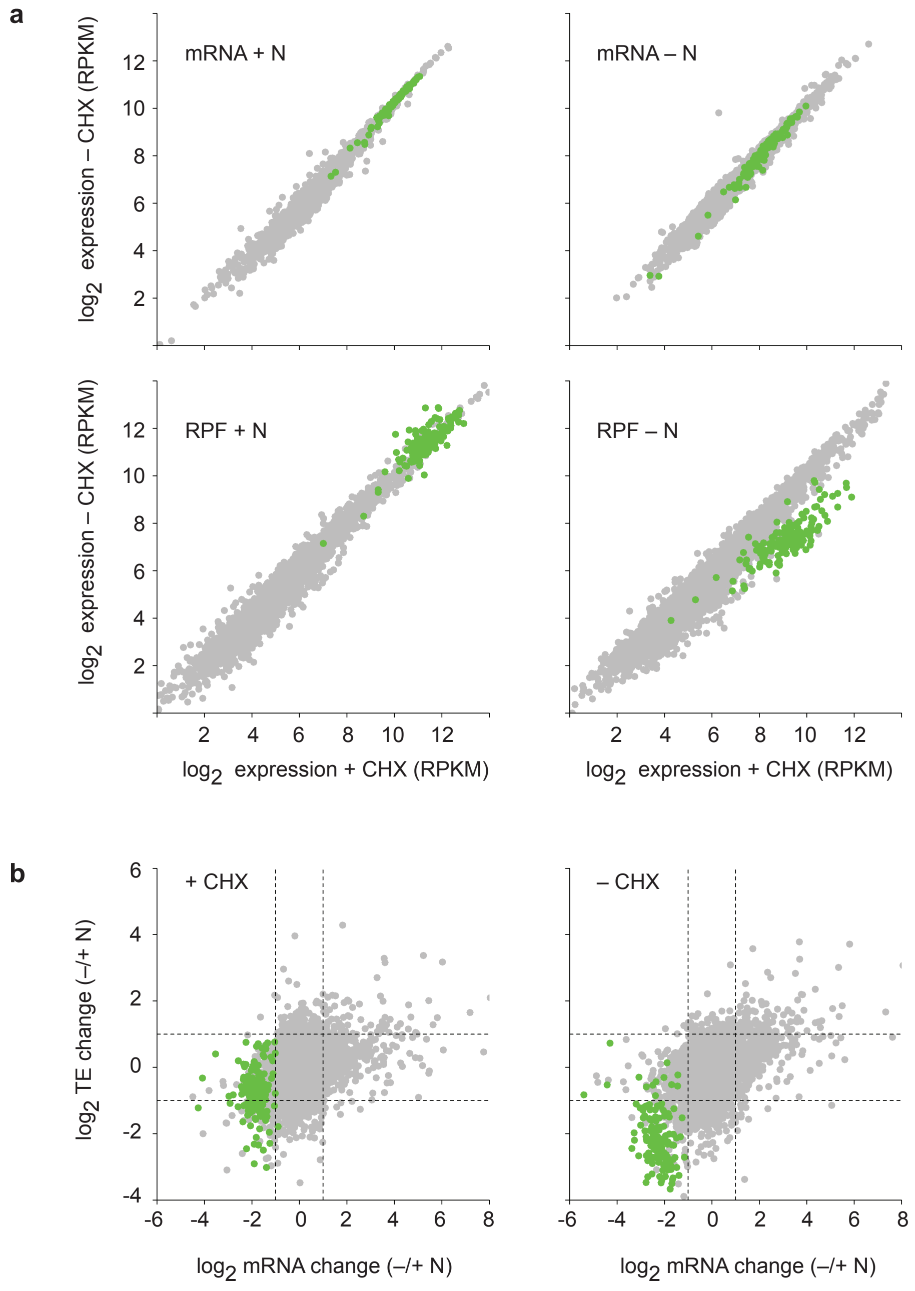

Supplementary Figure S1. Effects of CHX on overall ribosome densities on coding sequences (replicate 2).

a. Scatter plots comparing mRNA levels (top) and ribosome densities (bottom) between untreated and CHX-treated cells. Data are presented for cells growing in the presence of a nitrogen source (+ N) or starved for nitrogen (– N). Genes encoding ribosomal proteins are shown in green. All data have been normalized to RPKMs (Reads Per Kilobase per Million mapped reads). **b.** Comparison of changes in mRNA levels and ribosomal densities between cells starved for nitrogen (– N) and unstressed cells (+ N). Data are presented for CHX-treated cells (left) and for untreated cells (left). Genes encoding ribosomal proteins are shown in green.

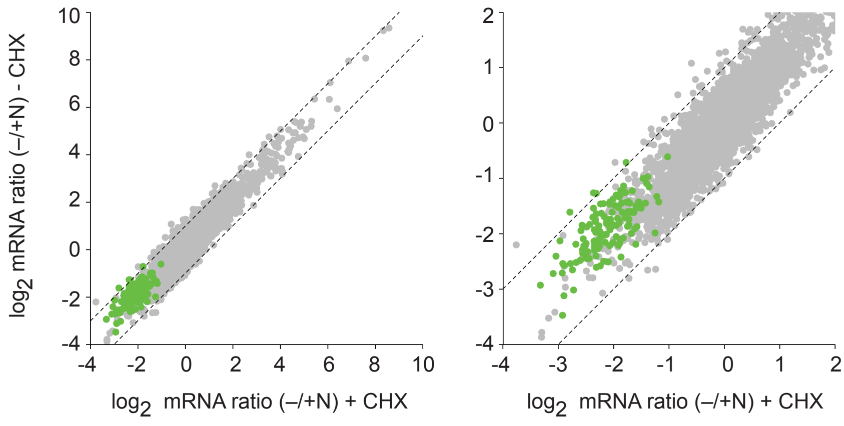

Supplementary Figure S2. Effects of CHX on changes in mRNA abundance upon stress.

Scatter plots comparing ratios of mRNA levels between nitrogen starved and control levels in CHX-treated and CHX-untreated cells. The dotted lines correspond to 2-fold changes. The plot on the left shows all ratios and that on the right zooms into the region containing the RP genes.

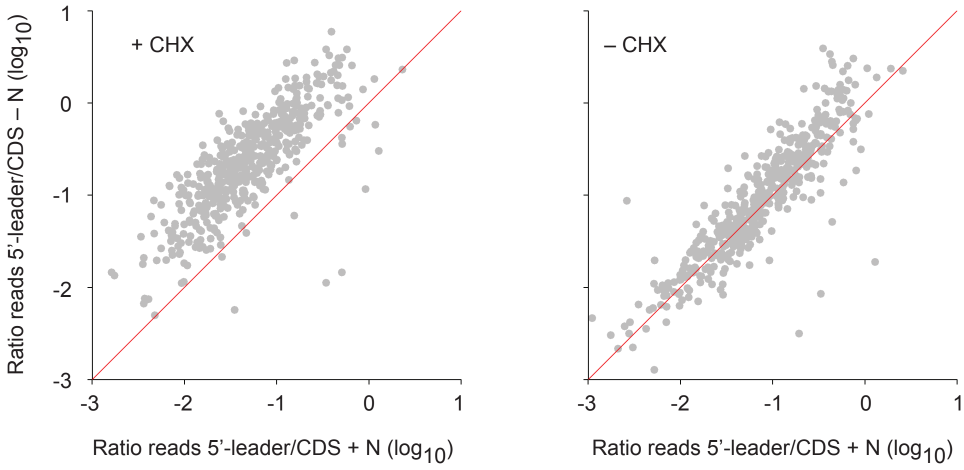

Supplementary Figure S3. Effects of CHX on ribosome densities on 5' leader sequences (replicate 2).

Scatter plot comparing the ratio of reads mapping to 5' leader sequences to reads mapping to coding sequences (CDS) for individual genes; each plot compares unstressed cells (+ N) and nitrogen starved cells (– N). Data are presented for cells treated with CHX (left) or untreated (right). The red lines correspond to a ratio of 1.

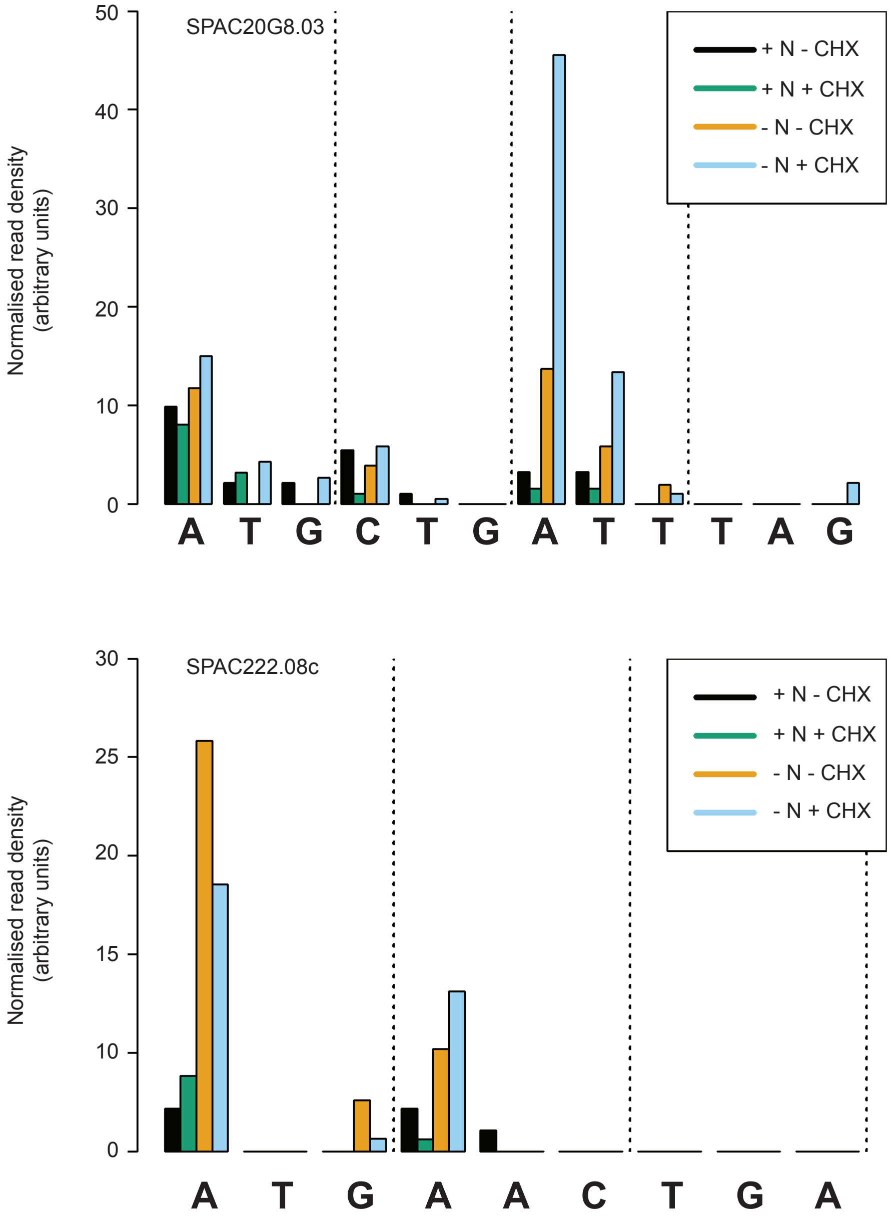

Supplementary Figure S4. Effects of nitrogen starvation and CHX on uORF translation.

Ribosomal densities in the presence and absence of nitrogen, and in CHX treated and untreated cells. Data are shown for uORFs of the indicated genes. Densities have been normalised for mRNA abundance and read depth. The sequences of the uORFs are indicated under the plots.

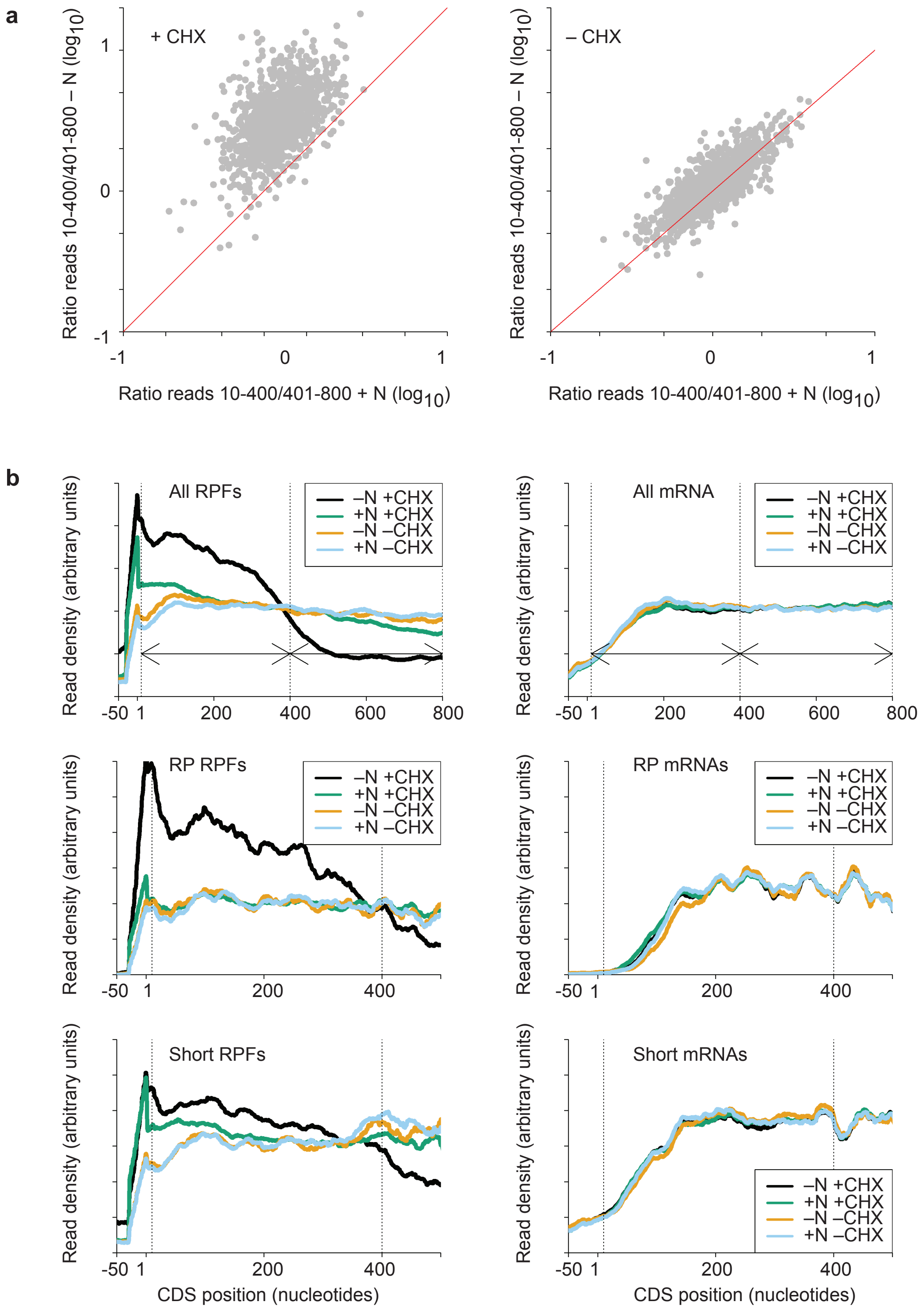

Supplementary Figure S5. Effects of CHX on ribosome distribution across coding sequences (replicate 2).

a. RPFs on nucleotides 10 to 400 and on nucleotides 401-800 are quantified in different experimental conditions, and the ratio between both numbers is calculated. Scatter plot comparing the ratios obtained as defined above for individual genes; each plot compares unstressed cells (+ N) and nitrogen starved cells (– N). Data are presented for cells treated with CHX (left) or untreated (right). The red lines correspond to a ratio of 1. **b.** Metagene displaying average distributions of RPFs along coding sequences in four experimental conditions. Data are shown for all genes, all ribosomal protein genes, and short genes (<600 nucleotides) excluding ribosomal protein genes. The graphs on the left correspond to ribosome protected fragments, and those on the right to mRNA-seq libraries. A running window of 30 nucleotides was used to smoothen the data. Vertical dashed lines correspond to positions 10, 400 and 800 nucleotides.

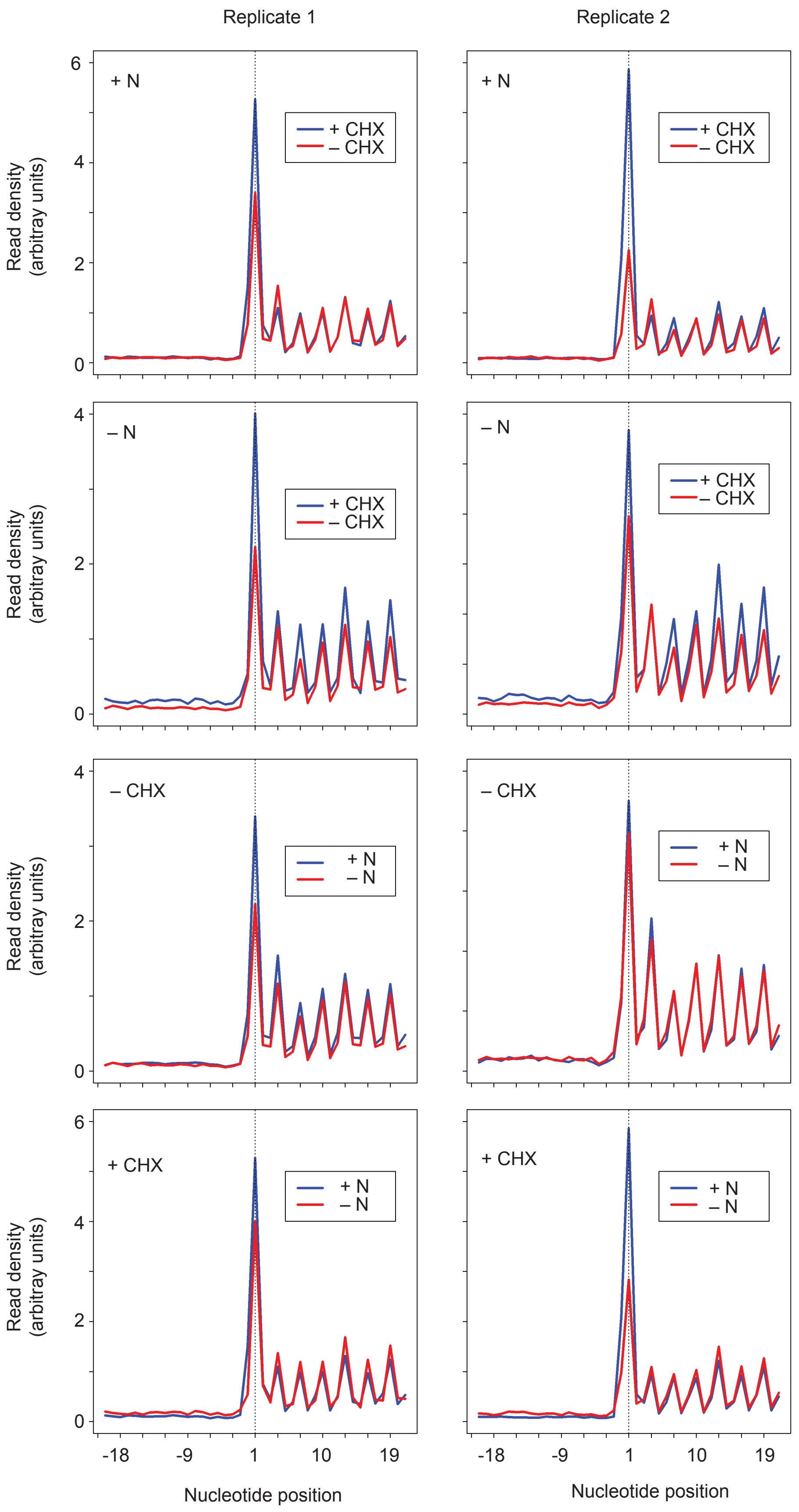

Supplementary Figure S6. Enrichment of ribosomes on initiation codons.

Metagene displaying average distributions of RPFs along coding sequences in regions around initiation codons. Each column corresponds to an independent replicate.

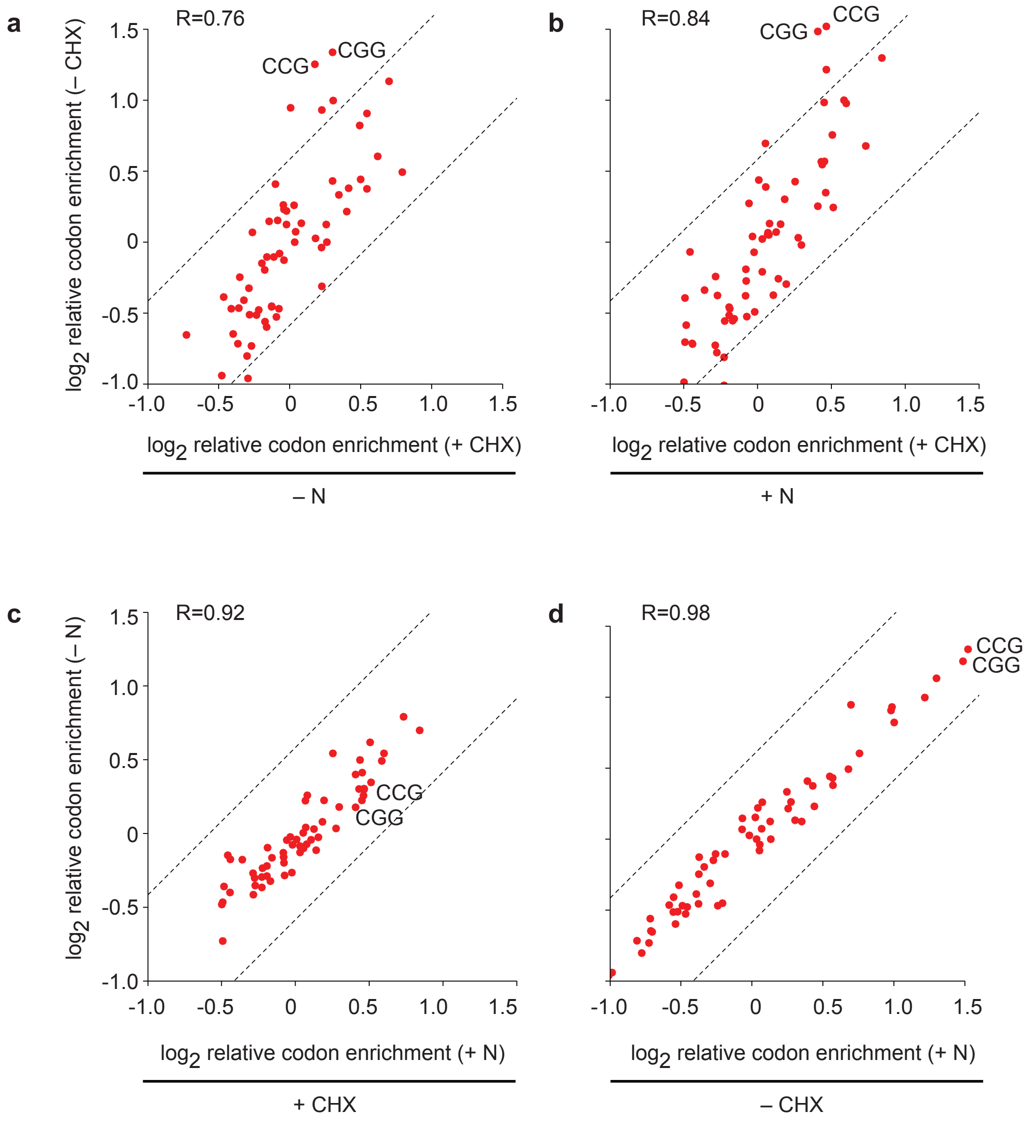

Supplementary Figure S7. Effects of CHX on relative codon occupancies (replicate 2).

Scatter plots displaying relative codon occupancies obtained as described in Methods. Each dot corresponds to a single codon. Termination codons are not displayed. The positions of rare codons CCG and CGG are indicated. The dotted lines correspond to 1.5-fold differences. The Pearson correlations between datasets are indicated. **a.** Comparison of the effects of CHX treatment in nitrogen-starved cells. **b.** As in A, for cells grown with a nitrogen source. **c.** Comparison of the effects of nitrogen starvation in the presence of CHX. **d.** As in C, in the absence of CHX.

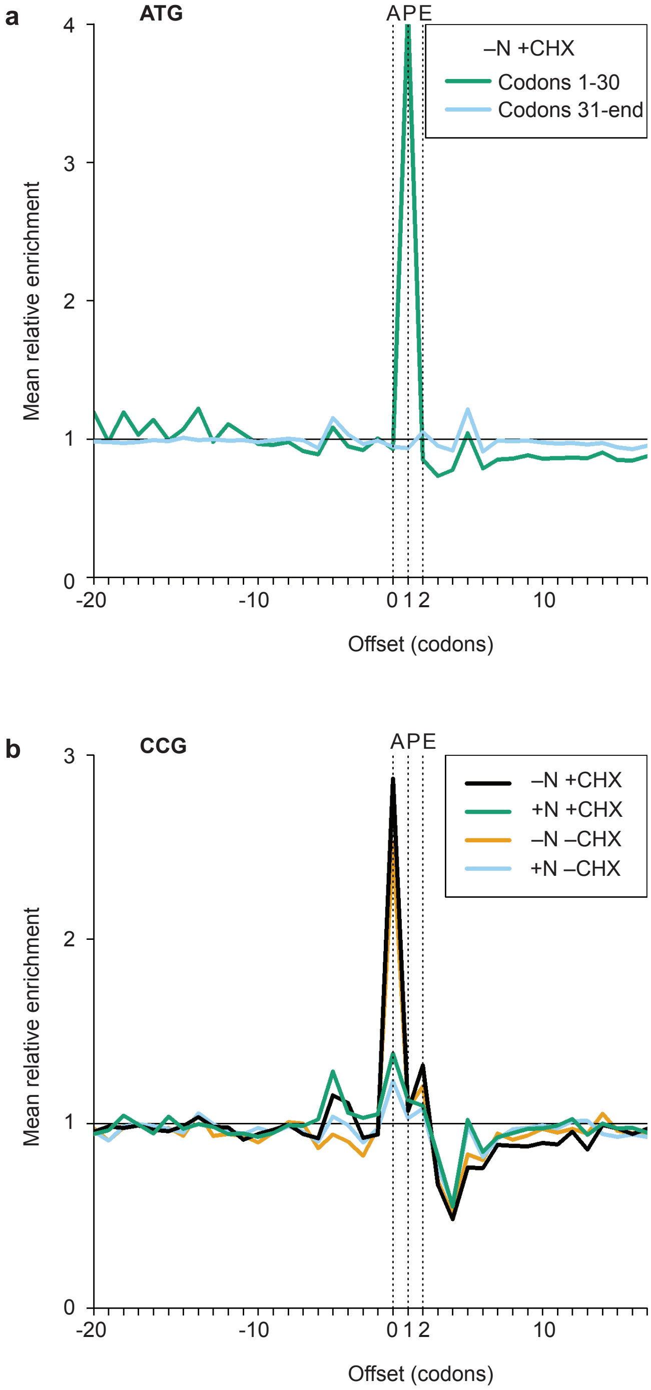

Supplementary Figure S8. Patterns of ribosome density around different common identities.

Profiles of mean relative codon enrichments (calculated as in *Hussman et al. PLoS Genetics 2015*) around the specified codons. Offsets of 0 and +1 correspond to the A- and P-sites, respectively. **a.** ATG codons when considering codons 1-30, or from codon 31 to the end of the sequence. Data are shown for nitrogen-starved, CHX-treated samples. Note that an enrichment is observed only when initiation codons are considered. **b.** CCG codons, considering codons 31 to the end of the CDS. Data are shown for ± nitrogen and ± CXH treatment.
